# New insights into long noncoding RNAs and pseudogenes in prognosis of renal cell carcinoma

**DOI:** 10.1186/s12935-018-0652-6

**Published:** 2018-10-11

**Authors:** Binghai Chen, Chengyue Wang, Jin Zhang, Yang Zhou, Wei Hu, Tao Guo

**Affiliations:** 1 Department of Urology, Affiliated Hospital of Jiangsu University, Jiangsu University, Zhenjiang, 212000 Jiangsu People’s Republic of China; 2 Department of General Surgery, Affiliated Hospital of Jiangsu University, Jiangsu University, Zhenjiang, Jiangsu People’s Republic of China; 3 Department of Andrology, The First Affiliated Hospital of University of South China, No. 69 Chuan Shan Road, Hengyang, 421001 Hunan People’s Republic of China

**Keywords:** Long noncoding RNA, Pseudogenes, Renal cell carcinoma, cBioPortal, Biomarker, Overall survival, Recurrence, PIK3CD-AS1, Signature

## Abstract

**Background:**

Increasing evidence suggests a critical role for long noncoding RNAs (LncRNAs) and pseudogenes in cancer. Renal cell carcinoma (RCC), the most common primary renal neoplasm, is highly aggressive and difficult to treat because of its resistance to chemotherapy and radiotherapy. Despite many identified LncRNAs and pseudogenes, few have been clearly elucidated.

**Methods:**

This study provides new insights into LncRNAs and pseudogenes in the prognosis of RCC. We searched an online database to interrogate alterations and clinical data on cBioPortal. We analysed LncRNA and pseudogene signatures to predict the prognosis of RCC based on a Cox model. We also found potential serum biomarkers of RCC and validated them in 32 RCC patients, as well as healthy controls.

**Results:**

Alterations were found in 2553 LncRNAs and 8901 pseudogenes and occurred in up to 23% of all cases. Among these, 27 LncRNAs and 45 pseudogenes were closely related to prognosis. We also identified signatures of LncRNAs and pseudogenes that can predict overall survival and recurrence of RCC. We then validated the relative levels of these LncRNAs and pseudogenes in the serum of 32 patients. Six of these, including LINC00520, PIK3CD-AS1, LINC01559, CEACAM22P, MSL3P1 and TREML3P, could be non-invasive biomarkers of RCC. Finally, we selected PIK3CD-AS1 to determine its role in RCC and found that upregulation of PIK3CD-AS1 was closely associated with higher tumour stage and metastasis.

**Conclusions:**

These signatures of LncRNAs and pseudogenes can predict overall survival and recurrence of RCC. LINC00520, PIK3CD-AS1, LINC01559, CEACAM22P, MSL3P1 and TREML3P could be non-invasive biomarkers of RCC. These data suggest the important roles of LncRNAs and pseudogenes in RCC, and therefore provides us new insights into the prognosis of RCC.

**Electronic supplementary material:**

The online version of this article (10.1186/s12935-018-0652-6) contains supplementary material, which is available to authorized users.

## Background

Only approximately 2% of genes in the human genome encode proteins. However, it is now widely accepted that approximately 80% of the human genome is functional, based on ENCODE data. This 80% contains regulatory elements, as well as noncoding RNA genes [1]. The widely discovered noncoding RNAs include miRNA (microRNA), LncRNA (long noncoding RNA), and pseudogenes [2–4].

Compared with widely known and studied miRNAs, the function and mechanism of LncRNAs and pseudogenes have not been elucidated [3, 4]. LncRNA is a noncoding RNA with more than 200 nucleotides. Increasing evidence shows characteristic abnormal expression of LncRNAs in many tumours [5]. LncRNAs regulate oncogenes and tumour suppressor genes, and thus affect the phenotype of cancer cells and biological behaviours including proliferation, differentiation, invasion, and angiogenesis [5]. On the other hand, pseudogenes that have similar DNA sequences to coding genes lost the original functions because of mutations [6]. A growing number of studies have shown that pseudogenes have important biological functions [7, 8]. Pseudogenes have been described as miRNA sponges and ceRNAs (competing endogenous RNAs) to regulate other genes. There are likely many additional currently unexplored mechanisms by which pseudogenes act [9]. Pseudogenes also induce endogenous small interfering RNAs to inhibit the expression of functional genes [4].

The role of LncRNAs and pseudogenes in renal cell carcinoma (RCC) has been reported but not yet fully elucidated [9]. RCC is the most common primary renal neoplasm. Worldwide studies have indicated an increasing incidence and mortality of RCC [10]. Approximately one-third of RCC patients present with advanced cancer at the time of diagnosis, and almost half of patients will develop RCC with metastasis [11]. In addition, patients with advanced RCC have poor prognosis, as RCC has shown resistance to chemotherapy and radiotherapy [12]. Thus far, valuable molecular markers of RCC for early diagnosis and prognosis are still controversial [13]. Thus, it is essential to have better understanding of RCC and develop new molecular markers.

In the present study, we analysed the months survival (MS) and months disease-free (MDF) of RCC combined with alterations of LncRNAs and pseudogenes. We also identified signatures of LncRNAs and pseudogenes and investigated how we can benefit from the signatures based on the data in the cBioPortal database [14]. We then validated the relative levels of these LncRNAs and pseudogenes in the serum of 32 patients. Our findings suggest that 6 of these can be non-invasive biomarkers of RCC. Among all the genes, PIK3CD-AS1 is the only one that is closely related to all of the important clinical features. We also found that PIK3CD-AS1 may promote metastasis based on characteristics of PIK3CD-AS1 in RCC.

## Methods

### Patients and blood samples

A total of 32 consecutive patients with RCC were included in the study. All of these patients were diagnosed based on biopsy of lymph nodes or postoperative pathological diagnosis. There was no history of urinary surgery, chemotherapy, or radiotherapy. We analysed the data, including gender, age, laterality, metastasis, lymph nodes, pathologic tumour stage, tumour pathologic PT, and volume of tumour. Thirty-two individuals who came for routine health examination were enrolled in this study, and they did not have any history of cancer. All blood samples were collected after all the patients signed consent document approved by the Ethics Committee of the Affiliated Hospital of Jiangsu University.

### Database search and gene signature selection

A total of 2772 LncRNAs and 12728 pseudogenes were downloaded from HGNC (http://www.genenames.org). A total of 2553 LncRNAs and 8901 pseudogenes were recognized by the cBioPortal after we input them one by one, as described previously [15]. We chose the kidney renal clear cell carcinoma (TCGA, provisional), as it has numerous cases compared with other datasets. The alterations of mutation, copy number alteration and expression of LncRNAs and pseudogenes were calculated separately. The P-values of MS and MDF were also determined based on the clinical data on cBioportal. We selected 27 LncRNAs and 46 pseudogenes in which expression was closely related to MS and MDF, with intact clinical data (Additional file 10: Table S9). Gene signature selection was based on the Cox model, as we described previously [15].

### Real-time PCR

Total serum RNA was extracted with TRIzol^®^ reagent (Thermo Fisher Scientific, Waltham, MA, USA.). Then, 0.5 µg of RNA was reverse transcribed to cDNA. The real-time PCR was performed with Brilliant SYBR Green Master mix (Bio-Rad Laboratories, Hercules, CA, USA) on a Roche LightCycler^®^ 480 Instrument II (Roche Applied Science, Mannheim, Germany), according to the protocol. The annealing temperature was 60 °C for 30 s. We used the 2^−ΔΔCt^ method to determine the relative level. GAPDH served as a normalizing control. All primers are included in Additional file 1: Table S11.

### Statistical method

The statistical analysis was performed with the SAS (version 9.3, the SAS institute). One-way analysis of variance was conducted with Tamhane’s T2 post hoc test to consider heterogeneity of variance using SPSS software (version 17, SPSS Inc., Chicago, IL). P-values were two-sided, and a value of 0.05 was considered to be statistically significant.

## Results

### Genetic alterations of LncRNAs in RCC

**Fig. 1:**
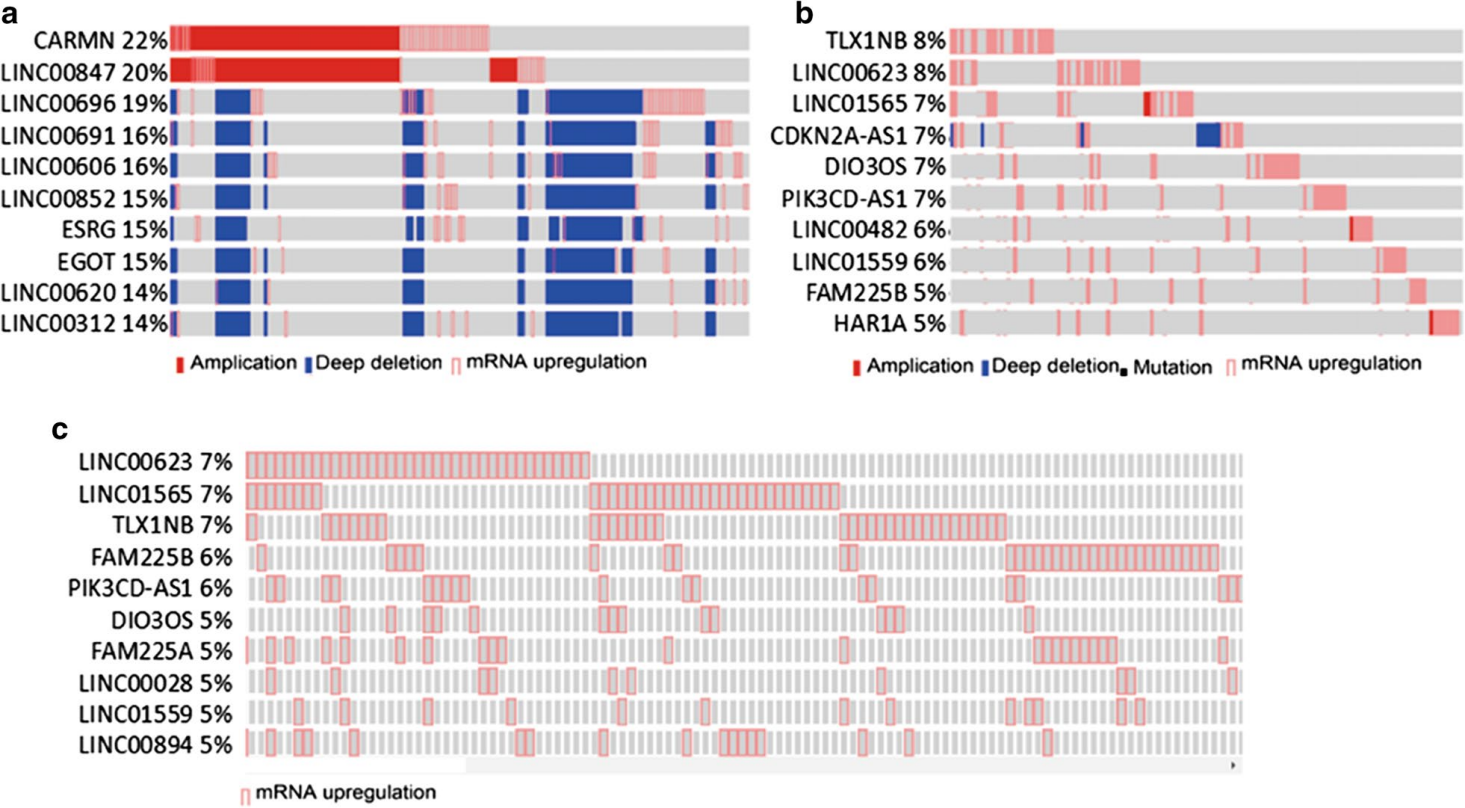
**a** The top 10 altered LncRNAs (including amplifications, deep deletions and mRNA expression). **b** The top 10 MS- and MDF-related LncRNAs. **c** The top 10 altered LncRNAs (mRNA expression only)

To uncover the roles of LncRNAs in RCC, we first downloaded all approved LncRNAs from HGNC. With the approved 2772 LncRNAs (Additional file 2: Table S1), we searched the cBioPortal database. A total of 2553 LncRNAs were recognized by the database, while the others were not included (Additional file 3: Table S2). We found obvious alterations of LncRNAs, including mutation, copy number alteration and expression. The total occurence of LncRNA alterations occurred in up to 22% of all cases (Additional file 4: Table S3). Among them, 10 LncRNAs (CARMN, LINC00847, LINC00696, LINC00691, LINC00606, LINC00852, ESRG, EGOT, LINC00620 and LINC00312) were altered in 46% of all cases with complete data. Amplifications, deep deletions and mRNA upregulations were included (Fig. 1a).

In our previous research, alterations of LncRNAs validated by cBioPortal were related to overall survival in breast cancers [15]. We thus tried to find the significantly altered LncRNAs in RCC based on the data in cBioPortal. The *P* value of months survival (MS) and that of months disease free (MDF) for all genes, alterations of which were over 2%, were analysed in the database (Additional file 4: Table S3). Among them, 27 LncRNAs were closely related to prognosis, according to the two P-values (Additional file 5: Table S4). Interestingly, the top 10 of the 27 LncRNAs were not as same as those in Fig. 1a, which suggests that only some of the LncRNA alterations were related to MS and MDF. The top 10 MS and MDF-related LncRNAs (TLX1NB, LINC00623, LINC01565, CDKN2A-AS1, DIO3OS, PIK3CD-AS1, LINC00482, LINC01559, FAM225B, and HAR1A) accounted for alterations in 37% of all cases (Fig. 1b). Considering the important role of LncRNA expression, we also analysed expression alterations of LncRNAs in all cases with mRNA data (Additional file 6: Table S5). The top 10 LncRNAs contributed to alterations in 33% of cases (Fig. 1c).

### Genetic alterations of pseudogenes in RCC

**Fig. 2:**
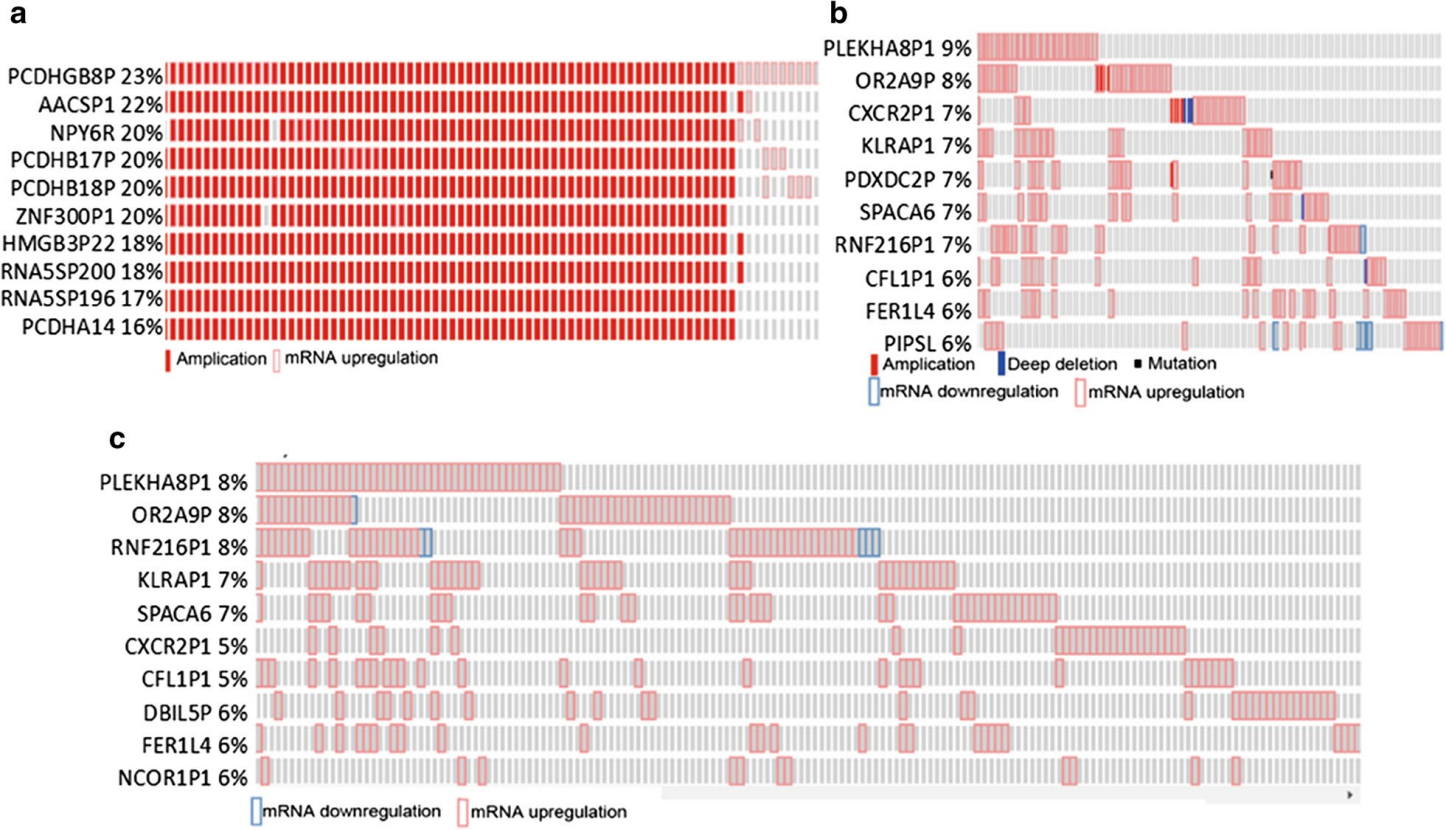
**a** The top 10 altered pseudogenes (including amplifications, deep deletions and mRNA expression). **b** The top 10 MS- and MDF-related pseudogenes. **c** The top 10 altered LncRNAs (mRNA expression only)

Pseudogenes are abundant in the human genome and are reported to regulate genes in a similar manner as LncRNAs. Thus, we also tried to determine the alterations of pseudogenes in RCC. Similarly, we downloaded 12728 pseudogenes (Additional file 2: Table S1), and 8901 of all pseudogenes were recognized by the database (Additional file 3: Table S2). We found alterations of pseudogenes in up to 23% of all cases (Additional file 7: Table S6). Among them, the top 10 altered pseudogenes accounted for 40% of all cases. The top 10 were PCDHGB8P, AACSP1, NPY6R, PCDHB17P, PCDHB18P, ZNF300P1, HMGB3P22, RNA5SP200, RNA5SP196 and PCDHA14 (Fig. 2a).

To find the relationship between pseudogenes and prognosis, we then investigated the two P-values in all pseudogenes, alteration of which were over 2%. Forty-five of all pseudogenes were significantly related to prognosis, according to the two P-values (Additional file 8: Table S7). The top 10 of the 45 pseudogenes, which were related to MS and MDF, comprised PLEKHA8P1, OR2A9P, CXCR2P1, KLRAP1, PDXDC2P, SPACA6P, RNF216P1, CFL1P1, FER1L4, and PIPSL. They contribute to alterations in 34% of all cases (Fig. 2b). Expression alterations of pseudogenes in all cases with mRNA data took place in 33% of cases (Fig. 2c) (Additional file 9: Table S8).

### LncRNAs significantly related to MS and MDF of RCC

**Fig. 3:**
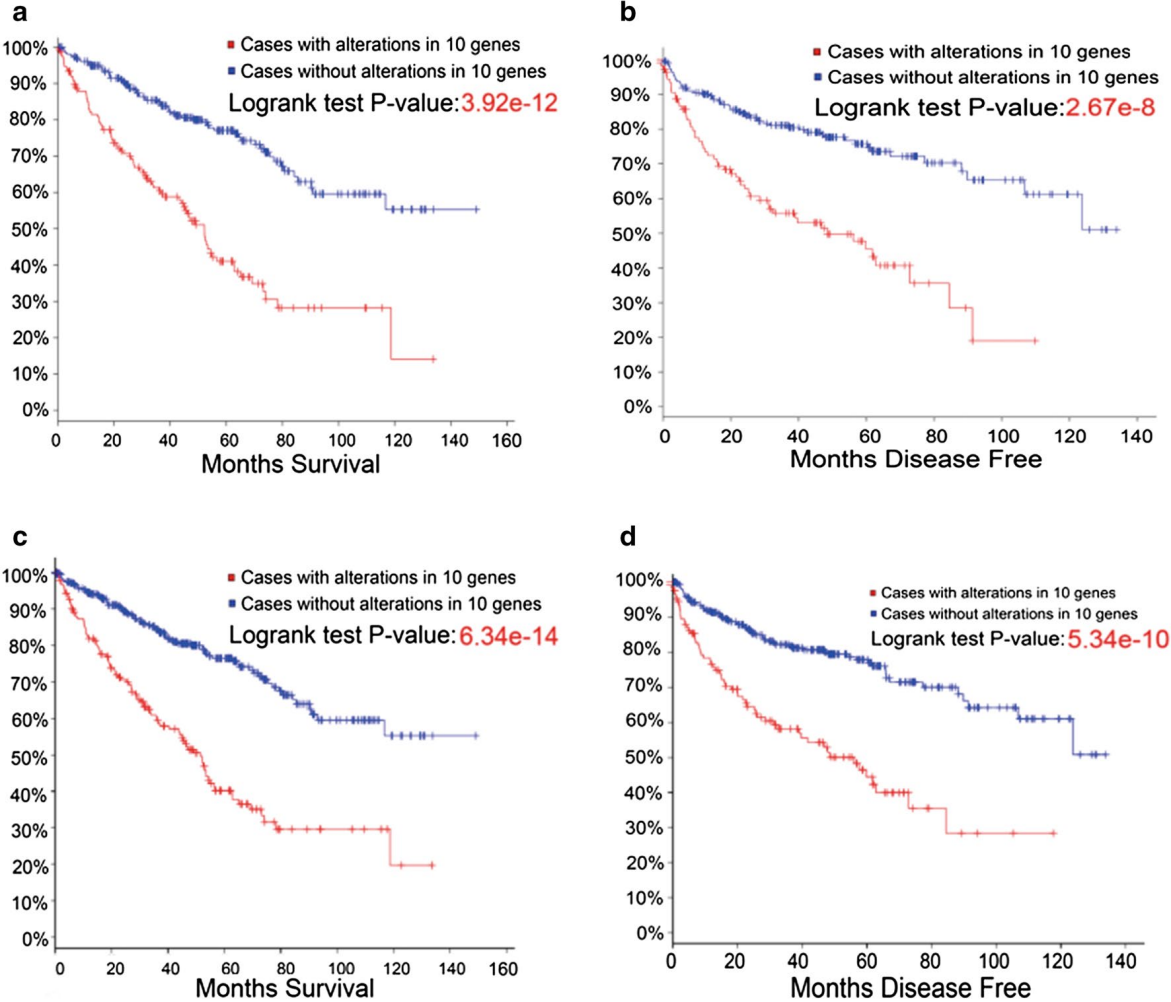
**a**, **b** The top 10 altered LncRNAs (including amplifications, deep deletions and mRNA) that significantly predicted MS and MDF. **c**, **d** The top 10 altered LncRNAs (mRNA only) that significantly predicted MS andMDF

When we combined the 10 LncRNAs, their significant associations with MS and MDF were observed. The log-rank test P-values for MS and MDF were 3.92e−12 and 2.67e−8, respectively (Fig. 3a, b). As shown in the cBioPortal database, the alterations of LncRNAs were composed of three parameters: mutation, copy number alteration (CNA) and expression. Considering that expression contributes the majority of alterations closely related to MS and MDF (Fig. 1b), we therefore checked the alteration of expression alone. We also calculated the P-values of both MS and MDF by combination of the 10 LncRNAs when expression was used as a sole criterion. The log-rank test P-values for MS and MDF (expression only) in LncRNAs were 6.34e−14 and 5.34e−10, respectively (Fig. 3c, d). Compared with those when we combined mutation, CNA and expression, the lower P-values resulting from expression alone suggest that alteration of expression of the LncRNAs might better predict the prognosis of RCC.

### Pseudogenes are also closely related to MS and MDF of RCC

**Fig. 4:**
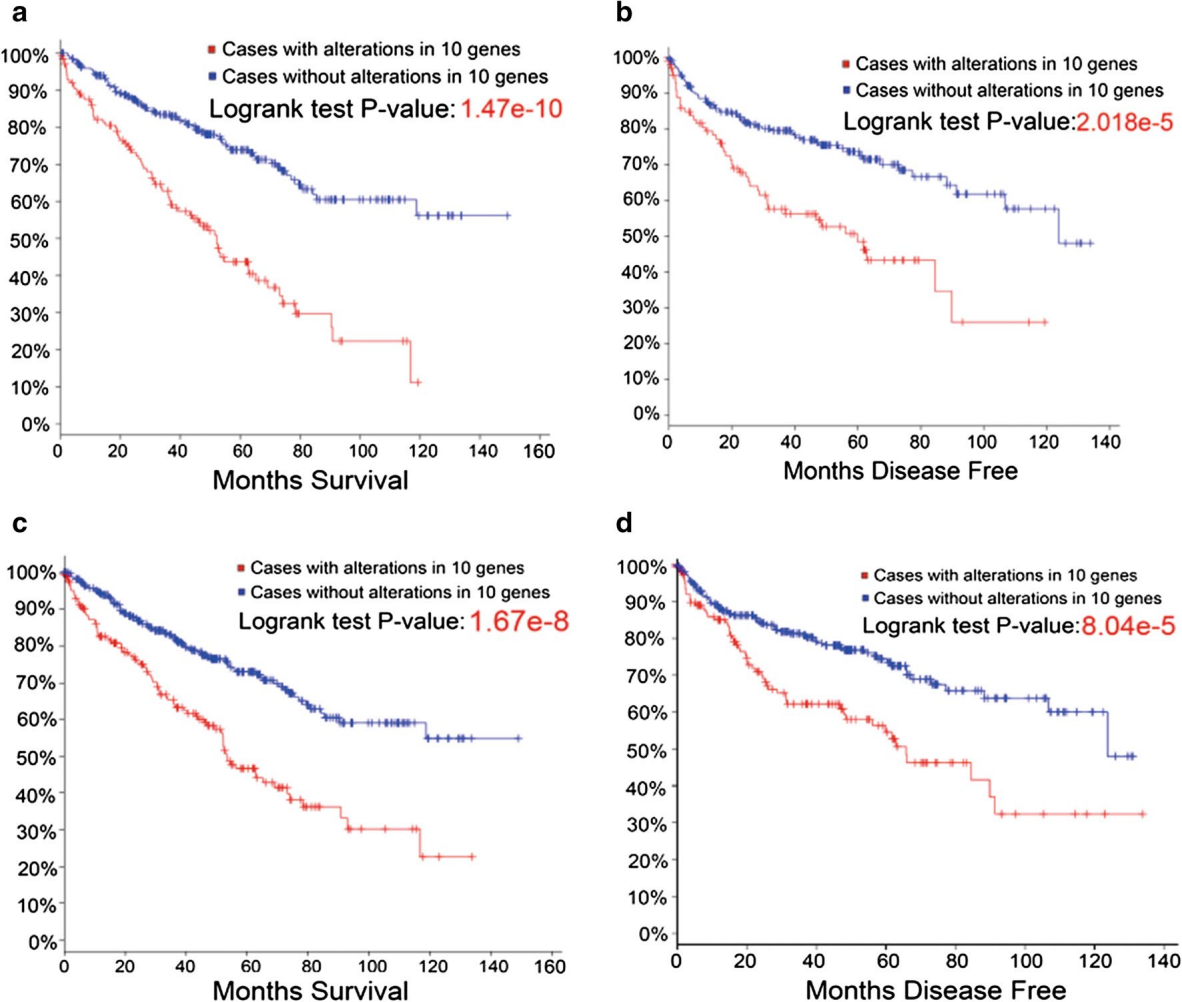
**a**, **b** The top 10 altered pseudogenes (including amplifications, deep deletions and mRNA) that significantly predicted MS and MDF. **c**, **d** The top 10 altered pseudogenes (mRNA only) that significantly predicted MS and MDF

When 10 pseudogenes were combined, we found that they had significant associations with MS and MDF. The log-rank test P-values in Fig. 4a, b were 1.47e−10 and 2.018e−5, respectively (Fig. 4a, b). Another search with alterations of gene expressions was also performed. We identified the P-values of both MS and MDF by combination of the 10 genes when expression was used as a sole criterion. The log-rank test P-values for MS and MDF (expression only) in pseudogenes were 1.67e−8 and 8.044e−5, respectively (Fig. 4c, d). These two P-values were much less than those with total alterations, suggesting that alteration of expression plays amore important role in predicting the prognosis of RCC.

### LncRNA and pseudogene signatures predicted overall survival and recurrence of RCC

**Fig. 5:**
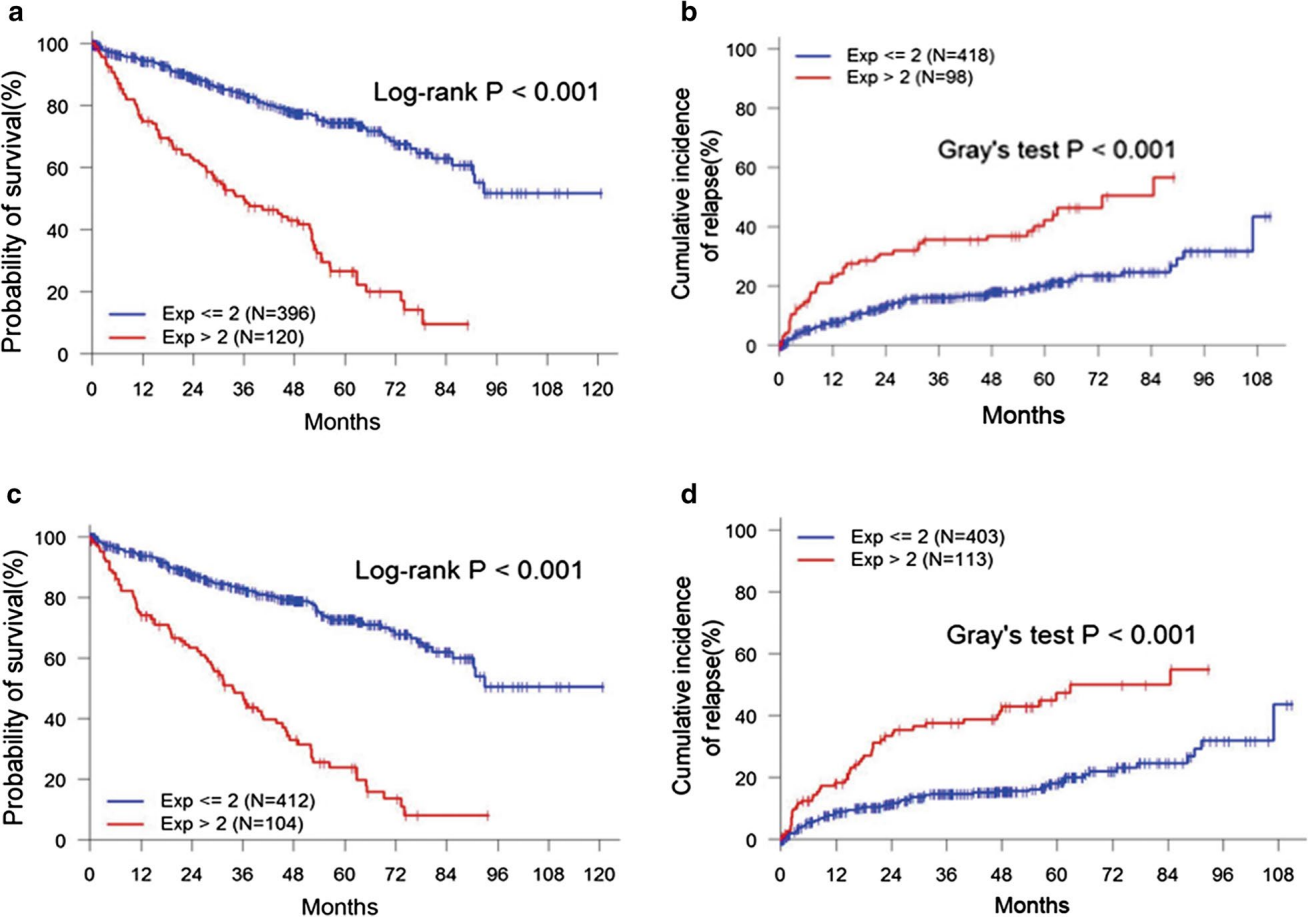
**a** The 8-LncRNA signature predicts the MS of RCC. **b** The 5-LncRNA signature predicts the MDF of RCC. **c** The 8-pseudogene signature predicts the MS of RCC. **d** The 7-pseudogene signature predicts the MDF of RCC

**Fig. 6:**
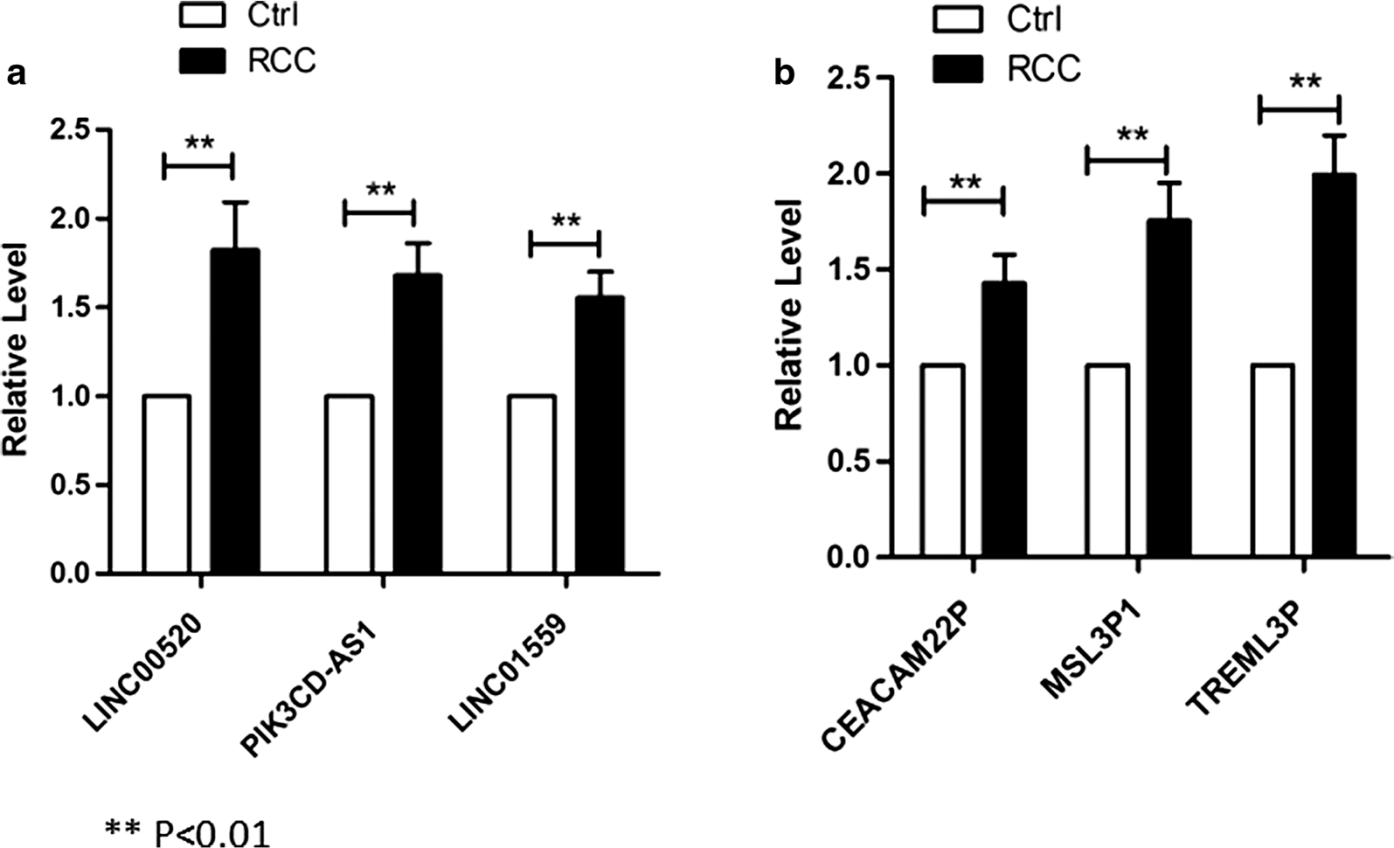
**a** Three LncRNAs (LINC00520, PIK3CD-AS1 and LINC01559) were overexpressed in the serum of patients with RCC. **b** Three pseudogenes (CEACAM22P, MSL3P1 and TREML3P) were overexpressed in the serum of the RCC patients. Values are mean ± SD

Thus far, there is no widely accepted signature that can be used to predict the prognosis of RCC. Therefore, we tried to include more clinical samples in the analysis of predictors in RCC using LncRNA and pseudogene signatures. We performed another search with the method we used previously. Of note, there were 27 LncRNAs and 46 pseudogenes, expressions of which are closely related to MS and MDF, with intact clinical data (Additional file 10: Table S9). Gene selections and signature installation were based on a Cox model. We included the LncRNAs or pseudogenes individually in the Cox model due to the MS or MDF. The variable with smallest P-value and below a 5% threshold entered the model. The model with smallest value in AIC was determined as the optimal model. According to the model, we identified 8 of 27 LncRNAs as a signature to predict the MS of RCC (Fig. 5a, Additional file 11: Figure S1A), and 5 to predict the MDF of RCC (Fig. 5b, Additional file 11: Figure S1B). Interestingly, LINC00520 and PIK3CD-AS1 can be used as predictors for both MS and MDF. On the other hand, 8 of 46 pseudogenes were believed to be predictors in the MS of RCC (Fig. 6c, Additional file 11: Figure S1C), while 7 pseudogenes were finally considered to predict the MDF of RCC (Fig. 6d, Additional file 11: Figure S1D). Thus, these signatures of LncRNAs and pseudogenes can help us to estimate the overall survival and recurrence of RCC more accurately and efficiently.

### Three LncRNAs and three pseudogenes are potential non-invasive biomarkers in RCC

There are no serum or urine biomarkers to diagnose RCC. We measured all LncRNAs and pseudogenes (included in Additional file 11: Figure S1) in the serum of 32 RCC patients as well as healthy controls. We found 3 LncRNAs (LINC00520, PIK3CD-AS1 and LINC01559) and 3 pseudogenes (CEACAM22P, MSL3P1 and TREML3P) overexpressed in the patients with RCC (Fig. 6). Other LncRNAs and pseudogenes could not be detected, or there were no significant differences. The altered serum levels of these LncRNAs suggested that these LncRNAs and pseudogenes might serve as promising biomarkers.

### Comparison with other signatures in RCC

It is reported that there are several gene signatures, including LncRNAs, coding genes, and miRNAs, in RCC. We then compared these signatures. A five serum-circulating LncRNA signature [16], including 5 LncRNAs, was analysed on cBioPortal. The analysis showed that these five LncRNAs were altered in only 54 (10%) of all cases, which suggests lower sensitivity compared with our signatures (Additional file 12: Figure S3). This signature predicts MS of RCC, and the P-value was 0.0318, which is much higher than those in our signatures. Furthermore, this signature could not predict the MDF (Table 1). This indicates that our signatures were more reasonable. We then investigated the signature of coding genes, including CKAP4, SLC40A1, OTOF, MAN2A2, and ISPD. These five coding genes were altered in 114 cases (Additional file 12: Figure S3). They can also predict the MS and MDF with P-values of 4.979e−4 and 9.883e−4, respectively (Table 1). Our signature was better than a three coding gene signature [17], which cannot predict the prognosis according to analysis on cBioPortal (Additional file 13: Figure S2). Another miRNA signature [18] comprised 5 miRNAs; however, the alteration of these five miRNAs occurred in only 4 cases. Thus, according to all these findings, we believe that our LncRNA and pseudogene signatures might be more convincing and dependable compared to other reported signatures.

**Table 1:** Comparison of our signatures with other reported signatures

	Serum-circulating LncRNAs	Coding genes	miRNA	LncRNAs	Pseudogenes
P-value of MS	0.0318	4.98E−04	0.00480	0	0
P-value of MDF	0.647	9.88E−04	2.43e−8	4.71E−14	1.11E−11

### Alterations of LncRNAs, pseudogenes and clinical features

To further study the clinical significance of LncRNAs and pseudogenes, we investigated the associations between clinical features and genes, which are related to MS and MDF (Additional file 10: Table S9). The sex, age, laterality, metastasis, lymph nodes, pathologic tumour stage, tumour pathologic PT, and volume were analysed, and their correlation with the alterations of LncRNAs and pseudogenes was investigated. Several LncRNAs and pseudogenes were related to the metastasis, pathologic tumour stage, and tumour pathologic PT (Additional file 14: Table S10). In contrast, we did not see any relationship between these genes and the other clinical features (data not shown). Importantly, we showed that the 4th, 15th and 16th genes among the 73 genes were much more significantly related to metastasis, pathologic tumour stage, and tumour pathologic PT, respectively (Table 2, P < 0.001).

**Table 2:** Alterations of LncRNAs and pseudogenes associated with clinical features

Gene	Cases with alteration	Cases without alteration	P-value of metastasis	P-value of stage	P-value of tumour pathologic PT
SPACA6P	35	499	0.06304	0.000003	0.000002
MYH16	23	511	0.03569	0.000161	0.000006
DLEU2	27	507	0.01669	0.000048	0.000006
PIK3CD-AS1	30	504	0.00076	0.000005	0.000007
LINC00520	16	518	0.18129	0.022667	0.000009
RPSAP52	7	527	0.61462	0.00639	0.000014
PLEKHA8P1	45	489	0.00347	0.000016	0.00005
HERC2P2	24	510	0.05581	0.000686	0.000203
NBEAP1	16	518	0.16166	0.000835	0.000222
LINC00623	38	496	0.01218	0.000005	0.000268
ZNF767P	27	507	0.11029	0.000142	0.000323
SNHG17	34	500	0.01181	0.000281	0.000375
ZNF436-AS1	21	513	0.3424	0.000779	0.00046
OR2A9P	40	494	0.04758	0.002167	0.000541
CLCA3P	21	513	0.55887	0.004363	0.000792
LINC00592	27	507	0.28987	0.026707	0.000974
LINC01559	28	506	0.00021	0.000024	0.002526
INGX	23	511	0.00014	0.000142	0.04373
RAET1K	22	512	0.0086	0.000536	0.00129
FKBP9P1	18	516	0.0016	0.000946	0.003459
CXCR2P1	27	507	0.00095	0.004867	0.033003

### The characteristic and potential role of PIK3CD-AS1 in RCC

**Fig. 7:**
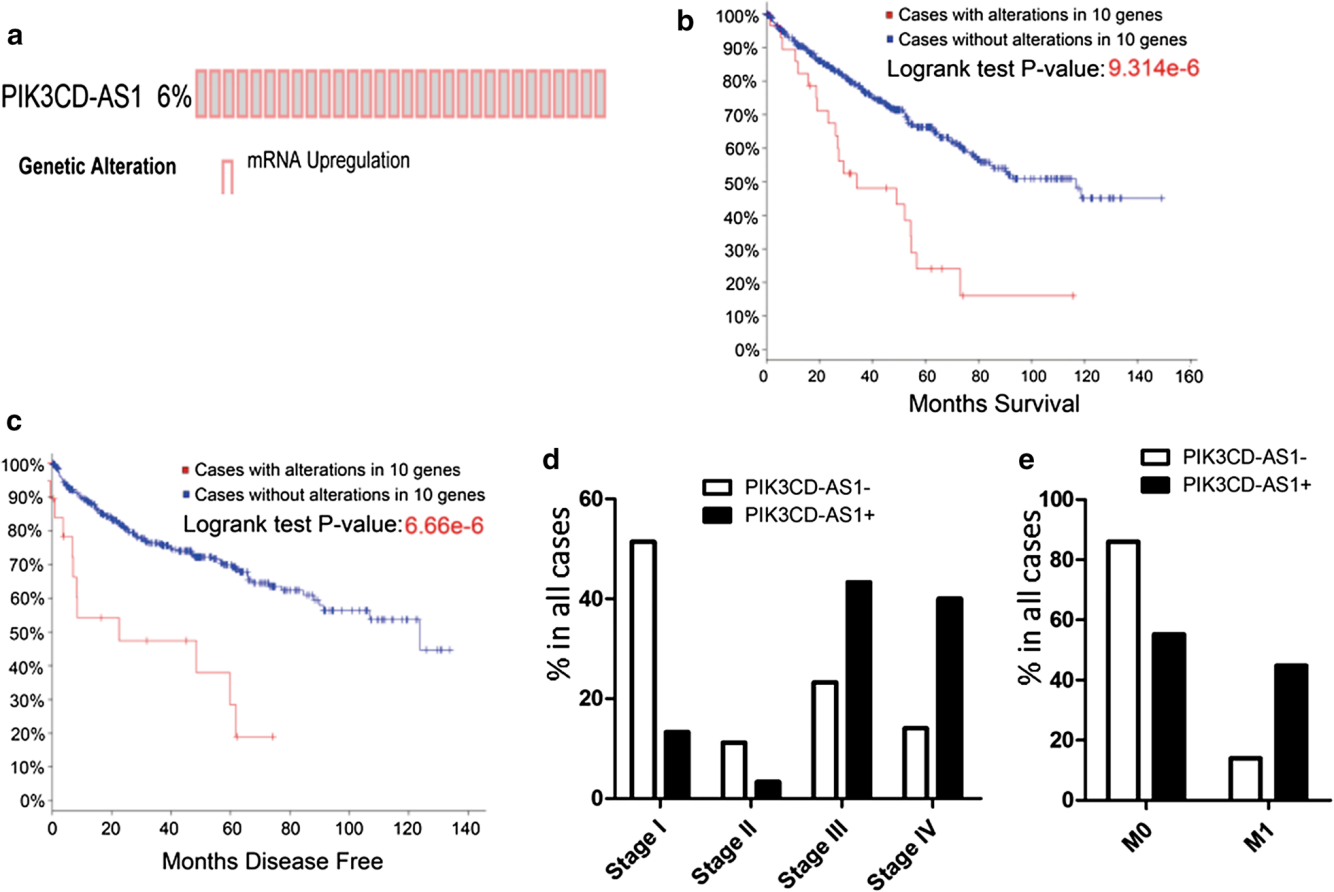
**a** PIK3CD-AS1 is upregulated in 6% of all cases. **b** and **c** PIK3CD-AS1 predicts MS and MDF of RCC, respectively. **d** Upregulation of PIK3CD-AS1 is closely associated with higher tumour stage. **e** Cases with upregulation of PIK3CD-AS1 have significantly higher incidence of metastasis

Among the 73 genes we discussed before, PIK3CD-AS1 was the only one that was closely related to all of the important clinical features. We tried to identify characteristics of PIK3CD-AS1 by another search on cBioPortal. Alterations of PIK3CD-AS1, which were all upregulations, accounted for 6% of all cases (Fig. 7a). The log-rank test P-values of PIK3CD-AS1 for MS and MDF were 9.314e−6 and 6.66e−6, respectively (Fig. 7b, c). Upregulation of PIK3CD-AS1 was closely associated with higher tumour stage, which has different treatments and suggests poor prognosis (Fig. 7d). Additionally, cases with upregulated PIK3CD-AS1 had significantly higher incidence of metastasis (44.83%) compared with that in cases without alternations (Fig. 7e). To uncover the potential role of PIK3CD-AS1, we searched cBioPortal again by simultaneous analysis of various crucial cell signal genes. We included cell cycle control, P53 signalling, DNA damage response, proliferation signalling, cell death regulation signalling, RTK signalling family, PI3K/AKT/mTOR pathway, RAS/RAF/MEK/ERK signalling, regulation of ribosomal protein synthesis, angiogenesis and invasion (Additional file 14: Table S10).

## Discussion

Few LncRNAs and pseudogenes are characterized, although increasing numbers of them have been identified. In addition, few are reported to be included in the signatures regarding the diagnosis and prognosis of RCC. We took advantage of the provisional database of cBioPortal, which includes the data for LncRNAs and pseudogenes, as well as clinical features.

The provisional dataset of RCC includes 538 cases and provides mRNA data in 534 cases, as well as complete data in 446 cases. In all cases, alterations of 2553 LncRNAs and 8901 pseudogenes, including mutation, copy number alteration and expressions, were investigated. We then found that some of the LncRNAs and pseudogenes were closely related to survival and recurrence. Among them, we included a few genes in the signatures based on the Cox model. These signatures are also characterized. First, all genes in the signature can separately predict the survival and recurrence of RCC; the signatures that combined the genes are considered to be of higher accuracy based on the P-values. Second, these signatures are based on the numerous sample dataset, as we mentioned before. Third, we have different signatures of LncRNAs and pseudogenes in prediction of overall survival and recurrence. Thus, these suggest that the signatures might work as potential prognostic markers and are worth further investigation.

Molecular biomarkers are currently investigated in RCC, and biomarkers for the therapy have not yet been clarified [19]. Previous studies focused on VEGF and cytokines. For example, clinical research of sorafenib suggested that VEGF works as an important molecular marker for progression-free survival and overall survival in advanced RCC cases [20]. It is reported that patients have a better prognosis if they have lower expression of interleukin 6 and hepatocyte growth factor [21]. On the other hand, another study showed the limits of cytokines in RCC [22]. For the other biomarkers, high levels of HIF-2a alone may indicate resistance to most of the targeted therapies [23].

Currently, increasingly more LncRNAs and pseudogenes are uncovered to be prognostic markers in human cancers. For instance, increased serum MALAT1 indicated a poor prognosis in gastric cancer. Further research has confirmed that knockdown of MALAT1 inhibited cell growth and invasion [24]. LINC01133 was considered as an inhibitor of EMT and metastasis by directly targeting SRSF6. Based on clinical study, LINC01133 may be a valuable biomarker and a therapy target worth further investigation [25]. On the other hand, increasing research suggests that pseudogenes play important roles in the pathogenesis and progression of cancer. Chen X uncovered the role of pseudogene CTNNAP1 and its cognate gene CTNNA1 in colorectal cancer [26]. Researchers in another study found that they benefited from INTS6P1 in plasma when identifying and screening hepatocellular carcinoma (HCC). Lower expression of plasma INTS6P1 was revealed in HCC. The authors suggested that INTS6P1 might be a valuable biomarker in HCC if the AFP were lower than 20 ng/ml [27].

Of note, in this study, we provide a number of LncRNAs and pseudogenes that can predict not only MS but also MDF. Based on the features of massive clinical cases from cBioPortal, 27 LncRNAs and 45 pseudogenes were selected after screening the entire database. They appeared to be closely related to both months survival and months disease free. Thus, these LncRNAs and pseudogenes are thought to be valuable prognostic markers in RCC, as alterations in them were determined in massive clinical samples. We also studied other clinical features besides prognosis. We focused on metastasis, pathologic tumour stage and tumour pathologic PT. According to this analysis, some genes were confirmed to be closely related to one of the three clinical features. Interestingly, PIK3CD-AS1 was selected, as it is the only one that is related to all three clinical features. PIK3CD-AS1 might be a promising LncRNA in RCC, as upregulation of PIK3CD-AS1 might increase the invasion ability and be related to poor prognosis. In addition, PIK3CD-AS1 might be involved in multiple biological processes, including P53 signalling, PI3K/AKT/mTOR pathway, and RAS/RAF/MEK/ERK signalling. Thus, this analysis provides us new insights into the mechanism of PIK3CD-AS1-related poor prognosis in RCC. We can begin with these signalling pathways before learning more details of the mechanism.

We defined four different signatures of LncRNAs and pseudogenes, which separately predict MS and MDF. Although these signatures of LncRNAs and pseudogenes in RCC have not been validated, their associations with cancer death or recurrence are clear. We input the serum-circulating LncRNA signature in this database and found that it was not related to MDF. Several possibilities may contribute to the conflicting results. First, this LncRNA signature is based on the serum samples rather than tissues, as in our signatures. Second, as long as any of the LncRNAs not related to MDF was added into the signature, it will significantly decrease the prediction ability. Finally, this signature was set to discriminate clear cell RCC (ccRCC) patients and healthy controls. There is not enough data and analysis to support the association with MS and MDF. Other signatures, including miRNA and coding genes, were also analysed in the database. Although they might work as predictions of MS and MDF, we found more benefit in our signatures of LncRNA and pseudogenes. First, the P-values of our signature were much lower than the others, suggesting that our signatures are more dependable. Second, the miRNA alterations in the signature were at a lower level; thus, the miRNA signature might be difficult to detect in most clinical cases, which hardly leads to an effective diagnosis and analysis. Third, the pseudogene signatures, which never been reported before, might introduce new methods to diagnose RCC by detecting them in the serum and urine. Thus, we further determined the level of these LncRNAs and pseudogenes in the serum. Although only a few of them were detectable in the serum and found to be significantly different, this result is of great interest based on the potential clinical roles of these LncRNA and pseudogene signatures. Therefore, the increased level of six LncRNAs and pseudogenes suggested a novel, effective, non-invasive method to diagnosis RCC.

Increasing evidence suggests that pseudogenes play important roles in cancers. For instance, alterations in the expression of OCT4 pseudogenes (OCT4-pg) in different cancers and pluripotent cell lines were observed [28]. In 2007, Lin [29] found that OCT4-pg could inhibit the growth and differentiation of mesenchymal stem cells. In human glioma and breast cancers, expression of OCT4-pg was not observed; however, expression of oct4-pg was confirmed and important roles were uncovered [7, 8, 30]. Kastler found that the pseudogene oct4-pg1 was a member of the Oct4 family and the only one that was expressed in prostate cancer cells. In addition, oct4-pg1, which encodes a protein containing 359 amino acids, maintains the unlimited proliferation and self-renewal of cancer cells. Pseudogenes regulate the expression of functional genes by competitive binding with miRNA to inhibit or promote the occurrence of cancer. For instance, pseudogene TUSC2p1 protects the expression of tumour suppressor gene TUSC2 by competitive binding with miRNA, and thus inhibits the proliferation of breast cancer cells [31].

In summary, this study provides a valuable solution for screening, considering increasing numbers of LncRNAs and pseudogenes. With this public dataset including vast clinical features, researchers can easily identify the LncRNAs and pseudogenes closely related to overall survival and disease-free months. Thus, researchers can focus on a few LncRNAs and pseudogenes with valuable clinical significance. The signatures that we found based on this dataset provide new insights into the diagnosis and prognosis of RCC. Finally, given that PIK3CD-AS1 is related to all three clinical features, we expect that it may be a special target of therapy in RCC.

## Conclusion

These signatures of LncRNAs and pseudogenes can predict the overall survival and recurrence of RCC. LINC00520, PIK3CD-AS1, LINC01559, CEACAM22P, MSL3P1 and TREML3P could be non-invasive biomarkers of RCC. These data suggest important roles for LncRNAs and pseudogenes in RCC, and therefore provide new insights into the prognosis of RCC.

## Additional files

**Additional file 1: Table S11.** All primers used in Real-time PCR.

**Additional file 2: Table S1.** All approved LncRNAs from HGNC.

**Additional file 3: Table S2.** A total of 2553 LncRNAs and 8901 of pseudogenes were recognized by the cBioPortal database.

**Additional file 4: Table S3.** Alterations of LncRNAs, including mutation, copy number alteration and expression.

**Additional file 5: Table S4.** 27 LncRNAs were closely related to prognosis.

**Additional file 6: Table S5.** Expression alterations of LncRNAs in all cases with mRNA data.

**Additional file 7: Table S6.** Alterations of pseudogenes, including mutation, copy number alteration and expression.

**Additional file 8: Table S7.** 45 pseudogenes were significantly related to prognosis.

**Additional file 9: Table S8.** Expression alterations of pseudogenes in all cases with mRNA data.

**Additional file 10: Table S9.** Expressions of LncRNAs and pseudogenes with intact clinical data.

**Additional file 11: Figure S1.** A) The 8-LncRNA signature that predicts the MS of RCC. B) The 5-LncRNA signature that predicts the MDF of RCC. C) The 8-pseudogene signature that predicts the MS of RCC. D) The 7-pseudogene signature that predicts the MDF of RCC.

**Additional file 12: Figure S3.** Other gene signatures, including LncRNAs, coding gene, and miRNAs, altered in RCC.

**Additional file 13: Figure S2.** The 3 LncRNAs in another signature are altered in 4% of all cases. B) and C) This signature cannot predict either MS or MDF of RCC.

**Additional file 14: Table S10.** LncRNAs and pseudogenes related to the metastasis, pathologic tumour stage, and tumour pathologic PT.

## Abbreviations

RCC: renal cell carcinoma
LncRNA: long noncoding RNA
miRNA: microRNA
MS: months survival
MDF: months disease free
CNA: copy number alteration
